# HSPB8 Facilitates the Oncogenesis and Advancement of Bladder Cancer via Activation of HSP27

**DOI:** 10.7150/jca.89994

**Published:** 2024-01-01

**Authors:** Wei Du, Kai Guo, Peng Wang, Jianye Zhong, Ning Jiang

**Affiliations:** Department of Urological Surgery, Zhujiang Hospital of Southern Medical University, Guangdong, Guangzhou, 510280, China.

**Keywords:** HSPB8, HSP22, HSP27, HSPB1, Bladder cancer

## Abstract

Bladder cancer (BCa) stands as a significant malignancy within the genitourinary system. Notably, heat shock proteins (HSPs) exhibit elevated expression in cells subjected to environmental stresses and have been linked to the progression of many human malignancies. Among these, the functional implications and specific mechanism of HSPB8 in BCa have yet to be fully explored. In this study, we measured HSPB8 expression in both BCa tissues and various cell lines, further delving into its influence on cellular behaviors. Our observations pinpoint an upregulation of HSPB8 in BCa, a trend strongly associated with more advanced clinical manifestations. Suppressing HSPB8 exhibited marked reductions in cell proliferation and migration capabilities, while simultaneously amplifying apoptosis and inducing cell cycle arrest. Reinforcing these findings, our in vivo analyses using mouse models showed similar trends. Notably, upon HSPB8 knockdown, levels of specific proteins including eNOS (S1177), Hsp27 (S78/S82), PRAS40(T246), RSK1/2(S221/S227), and STAT3 (S727) decreased, with Hsp27 (S78/S82) and PRAS40(T246) experiencing the most profound drops. Furthermore, the application of an HSP27 inhibitor effectively reversed the phenotypes caused by increased HSPB8 expression. Collectively, our results suggest that elevated HSPB8 expression could act as a potential prognostic marker for BCa, and targeting HSPB8 might open new therapeutic avenues for treating this malignancy.

## Introduction

Bladder cancer (BCa) ranks among the predominant malignant diseases afflicting the genitourinary system. Roughly 30% of BCa cases manifest as muscle-invasive bladder cancer (MIBC). This type characteristically exhibits a propensity for progression and metastasis. Distressingly, the 5-year survival rate for metastatic bladder cancer patients hovers around a mere 13 to 15 months 1. Those diagnosed with muscle-invasive bladder cancer frequently undergo radical cystectomy and urinary diversion, interventions that drastically alter urinary physiology and subsequently diminish post-operative life quality. Over the past three decades, little headway has been made in clinical approaches to bladder cancer treatment. Our deepened comprehension of bladder cancer biology, combined with prolific high-throughput sequencing initiatives, has heralded a clinical inclination towards targeted therapies and potent immunotherapies. A thorough grasp of the molecular mechanisms underpinning bladder cancer progression is pivotal for pinpointing prospective drug targets.

All cells invariably confront a myriad of environmental stressors, and a pronounced upregulation of heat shock proteins (HSP) emerges as a fundamental adaptive response. HSPB is identified as one of six distinct HSP family members, categorized primarily based on their molecular weights 2. HSPB proteins exhibit a relatively diminutive subunit molecular weight, ranging from 12-43kDa. Within this HSPB family, HSPB8, alternatively termed HSP22, commands particular attention 3. HSPB8 is a representative member of the intrinsic disordered protein (IDP) cluster, renowned for its hallmark structural versatility and adaptability 3.

Concurrently, HSPB8 appears to influence cellular proliferation and apoptotic pathways. Notably, HSPB8 overexpression is discerned in specific cancer types, especially those exhibiting elevated metastatic prowess, a scenario often correlated with a grim prognosis. Silencing of HSPB8 notably curtails MCF-7 cell proliferation and negates the proliferative impetus of 17β-estradiol, intimating a probable role for HSPB8 in the proliferative and migratory mechanisms governing breast cancer 4. Intriguingly, HSPB8 intrinsically bolsters radio sensitivity both in vitro and in vivo. Its absence in cyclin D1-overexpressing cells mitigates some cyclin D1-induced effects 5. Furthermore, HSPB8 can orchestrate the expression of oncogenic proteins via mRNA modulation. HSPB8 selectively engages with RBP Sam68, a factor central to RNA transport and modulation, thereby thwarting its function. This culminates in aberrant expression patterns for RRE-mediated and constituent transport element-dependent reporter genes 6. By modulating gene expression, HSPB8 potentially governs the trajectories of oncogenic and tumor-suppressive proteins, dictating cancer evolution and progression 7. A contemporaneous bioinformatics analysis ascertained an association between HSPB8 and immune cell infiltration in bladder cancer, suggesting its immune biomarker potential 8. Yet, the precise biological function and molecular mechanism of HSPB8 in BCa remain enigmatic, underscoring the need to elucidate its influence on BCa progression.

## Materials and methods

### Patients and tissue specimens

Bladder cancer specimens and corresponding adjacent non-cancerous tissues were sourced from Shanghai Outdo Biotech Co., Ltd. (Shanghai, China). Our study encompassed 108 samples from 68 distinct patients, comprising 68 cancerous and 40 normal tissues. Comprehensive clinical data were gathered and documented for each patient. All participating patients provided informed consent for tissue collection. The study was granted ethical clearance by the Medical Ethics Committee of Zhujiang Hospital of Southern Medical University.

### Cell lines and maintenance

For our research, human BCa cell lines T24 and EJ were sourced from the American Type Culture Collection (ATCC). Both T24 and EJ cells were cultivated in RPMI 1640 medium, enriched with 10% fetal bovine serum (FBS) and 1% penicillin-streptomycin solution. The cells were consistently housed in an incubator set at 37.0 °C with a 5% CO_2_ atmosphere. The culture medium was refreshed bi-daily. To verify the cell lines' authenticity, short tandem repeat (STR) profiling was employed.

### Immunohistochemical Analysis

Bladder cancer and adjacent tissue sections underwent deparaffinization, followed by antigen retrieval using 1× EDTA (Beyotime Biotechnology Co., Ltd, Shanghai, China). Subsequently, sections were blocked with 3% H_2_O_2_ for 10 min. The samples were then treated with primary HSPB8 antibody (1:200, Abcam, #ab151552) and left to incubate overnight at 4°C. Post-incubation, the primary antibody was removed with TBST washes, and sections were exposed to the secondary antibody (goat anti-rabbit IgG H&L: 1:400, Abcam, #97080) for 30 min at ambient temperature. DAB staining (Aidisheng, #ADS053W0, Jiangsu, China) was applied and monitored under a microscope, terminating within 10 min. The sections were counterstained with hematoxylin (Baso Diagnostics Inc., Zhuhai, China) for a brief 10-15 s duration. Neutral resin (China National Pharmaceutical Group Co., Ltd, Beijing, China) was used to mount the slides. All captured images were acquired using an optical microscope. Two independent pathologists assessed each slide. For quantitative evaluation, the IHC score was employed. Staining coverage was categorized as 0 (0%), 1 (1-25%), 2 (26-50%), 3 (51-75%), or 4 (76-100%). Staining intensity ranged from weak (0) to strong (3). Based on the combined scores of intensities and coverage, specimens were grouped as negative (0), positive (1-4), ++ positive (5-8), or +++ positive (9-12).

### Lentiviral RNAi Construction and Infection Procedure

Three RNA interference sequences were designed targeting the HSPB8 gene: TCTGGCAAACATGAAGAGAAA, TTGGAGAGAGCAGTTTCAACA, and CAAATCAAACCTATCCAACAA. Single-stranded DNA oligos representing these sequences were synthesized and annealed to produce double-stranded DNA. This DNA was then ligated into the lentiviral vector BR-V108 using restriction sites. Successful ligation products were transformed into competent E. coli cells, and positive clones were confirmed by PCR. For viral production, 293T cells were cotransfected with the lentiviral constructs along with the packaging plasmids pMD2. G and pSPAX2. Post 48 and 72 hours of transfection, the viral-laden supernatant was harvested, concentrated, and purified. T24 and EJ cells, in their logarithmic growth phase, were infected using 20 μl of the concentrated lentivirus (1 × 10^8^ TU/mL) in 6-well plates seeded with 2 × 10^5^ cells/well. Transduction efficiency was assessed visually after 48 hours, based on green fluorescent protein expression. Finally, the success of the RNAi-mediated knockdown was evaluated using real-time PCR and western blotting.

### RNA Extraction and Quantitative RT-PCR

Total RNA from cells was isolated using TRIzol reagent (Sigma, #T9424-100m) according to the manufacturer's protocol. The extracted RNA was reverse-transcribed to cDNA using the HiScript Q RT Supermix for qPCR (+gDNA WIPER) Kit (Vazyme, #R123-01, Nanjing, Jiangsu, China). Subsequently, quantitative PCR was performed employing the AceQ qPCR SYBR Green master mix Kit (Vazyme, #Q111-02, Nanjing, Jiangsu, China) in a reaction volume of 10 μl. The relative expression of RNA was quantified using the 2^-△△Ct^ method, with GAPDH serving as a reference gene. The primer sequences used were: HSPB8: Forward: 5'-ACCAAAGATGGATACGTGGAGG-3', Reverse: 5'-TGGGGAAAGTGAGGCAAATACT-3', GAPDH: Forward: 5'-TGACTTCAACAGCGACACCCA-3', Reverse: 5'-CACCCTGTTGCTGTAGCCAAA-3'.

### Western Blot Analysis

Post lentivirus infection, cells in stable growth were harvested for protein extraction using the BCa protein assay kit (HyClone-Pierce, #23225). Proteins were resolved on a 10% SDS-PAGE and transferred onto PVDF membranes. Membranes were blocked with 5% skim milk in TBST for 1 hour, incubated with primary antibodies overnight at 4°C, and then with secondary antibodies for 1 hour. After washing thrice with TBST, protein bands were visualized with the Immobilon Western Chemiluminescent HRP Substrate kit (Millipore, #RPN2232) and captured using a chemiluminescence imager. Primary antibodies included HSPB8 (1:1000, Proteintech, 15287-1-AP), PRAS40 (1:1000, Abcam, #ab151719), p-PRAS40 (1:1000, Abcam, # ab134084), HSP27 (1:1000, Abcam, #ab109376), p-HSP27 (1:1000, Abcam, # ab155987), and GAPDH (1:30000, Proteintech, #60004-1-lg). Secondary antibodies were goat anti-mouse (1:3000, Beyotime, #A0216) and goat anti-rabbit (1:3000, Beyotime, #A0208).

### HCS cell proliferation assay

Cells from either the shCtrl or shHSPB8 group (T24 and EJ) were digested, resuspended, and seeded in a 96-well plate at a density of 2000 cells/well with 100 µl of cell suspension. They were incubated at 37°C with 5% CO_2_. Using the Celigo image cytometer (Nexcelom Bioscience, Lawrence, MA, USA), cell images were captured daily for five consecutive days. Each group was assayed in triplicate. Based on the data, a 5-day cell proliferation curve was plotted.

### Wound healing assay

T24 and EJ cells from either the shCtrl or shHSPB8 group were seeded in a 96-well plate at a density of 5 × 10^4^ cells/well. Upon reaching 90% confluence, cells were exposed to a low concentration of FBS (Ausbian, #A11-102), and a scratch was made on the cell layer. Cells were subsequently incubated in 0.5% FBS medium at 37°C with 5% CO_2_. At specific time points (0, 48, or 72 h), scratch images were captured using Cellomics (Thermo, #ArrayScan VT1) to evaluate cell migration rates. This assay was conducted in triplicate.

### Transwell assay

Initially, the upper chamber was conditioned with 100 µl serum-free medium for 1-2 h. Cell suspensions of T24 and EJ cells from either the shCtrl or shHSPB8 groups were prepared in serum-free medium. 100 µl of this suspension was added to each chamber. Separately, the lower chamber was loaded with 600 µl of medium enriched with 30% FBS. The upper chamber was then positioned over the lower one and left to incubate for 24 h. Following incubation, cells were stained using 400 µl of Giemsa. Migrated cells from the upper chamber were visualized, photographed, and quantified under a microscope. This experiment was executed three times to gauge the cells' migration capability.

### Apoptosis Detection and Fluorescence-Activated Cell Sorting (FACS)

T24 and EJ cells from either shCtrl or shHSPB8 groups were cultured in 6-well plates. Upon reaching 70% confluence, cells were centrifuged at 1300 rpm for 5 minutes, discarding supernatants. Cells were then washed with precooled 4°C D-Hanks balanced solution (pH 7.2-7.4) and stained with Annexin V-APC (eBioscience, #88-8007) in the dark for 10 minutes, followed by the addition of Propidium Iodide (PI) for further staining. Apoptosis levels were assessed using a FACSCalibur (Millipore, #Guava easyCyte HT), and the apoptotic rate was analyzed. For cell cycle evaluation, T24 and EJ cells post-lentivirus infection were cultured in 6 cm dishes (5 mL/well). After similar processing, cells were washed with precooled 4°C PBS, followed by ethanol. Propidium iodide staining was done to ascertain cell cycle alterations through FACSCalibur (Millipore, #Guava easyCyte HT). Each procedure was replicated thrice.

### Human Phospho-Kinase Array-Membrane

To gauge the influence of HSPB8 knockdown on phospho-kinases, a Human Phospho-Kinase Array Kit (R&D, #ARY003C) was employed on T24 cells. Cells were first washed with PBS and subsequently resuspended in lysis buffer. Each well received 1 mL of lysate, followed by overnight incubation at 4°C. Handling Array membranes were washed three times with 1× Wash Buffer, each wash lasting 10 minutes. Post-wash, cells were exposed to 1 mL of Detection Antibody Cocktail for 2 hours at ambient temperature. Membranes underwent another series of washes as described earlier, then treated with streptavidin-HRP for half an hour at room temperature. To conclude, Chemi Reagent Mix was added, and the membrane signals were captured via a chemiluminescence imaging system.

### Nude mouse tumor formation model construction

Four-week-old female BALB/c nude mice were procured and the animal experiments secured approval from the Medical Ethics Committee of Zhujiang Hospital, Southern Medical University. For xenograft models, T24 cells from either the shCtrl or shHSPB8 group were employed, and each group consisted of 10 mice. T24 cells in the logarithmic growth phase were trypsin-digested and resuspended in PBS to prepare a cell suspension. Subsequently, 200 µl of this cell suspension, equivalent to 2 × 10^6^ cells, was subcutaneously injected into the mice. Throughout the feeding period, tumor dimensions were gauged using a Vernier caliper. On the final feeding day, mice received an intraperitoneal injection of 0.7% sodium pentobarbital, dosed at 10 µl/g. Subsequently, fluorescence intensity was assessed using an in vivo imaging system (Berthold Technologies, #LB983). After a span of 28 days, the mice were euthanized via cervical dislocation. The excised tumors were weighed, flash-frozen in liquid nitrogen, and preserved at -80°C.

### Ki-67 staining

Tumor tissue sections from mice were fixed in 4% paraformaldehyde containing 0.3% Triton X-100 at ambient temperature. For enhanced antigen retrieval, EDTA solution (Beyotime, #P0085) was employed. A 3% hydrogen peroxide solution was applied for 10 minutes to neutralize endogenous peroxidase. Sections were then incubated with the primary Ki-67 antibody (1:200, Abcam, #ab16667) at 4°C overnight. This was followed by a 2-hour room temperature incubation in the dark with the secondary antibody: goat anti-rabbit IgG H&L (HRP) (1:400, Abcam, #ab97080). Subsequently, sections were stained using hematoxylin (Baso, Zhuhai, Guangdong, China) and visualized under a microscope.

### Statistical analysis

Data analysis was performed using GraphPad Prism 7 (San Diego, CA). All results are depicted as mean ± SD. To determine statistical disparities, the unpaired t-test was applied. A p-value below 0.05 was deemed statistically significant. To discern the correlation between HSPB8 expression and the pathological attributes of BCa patients, both Mann-Whitney U and Spearman analyses were utilized. All experimental procedures were replicated three times.

## Results

### Elevated expression of HSPB8 was associated with BCa progression

**Figure 1:**
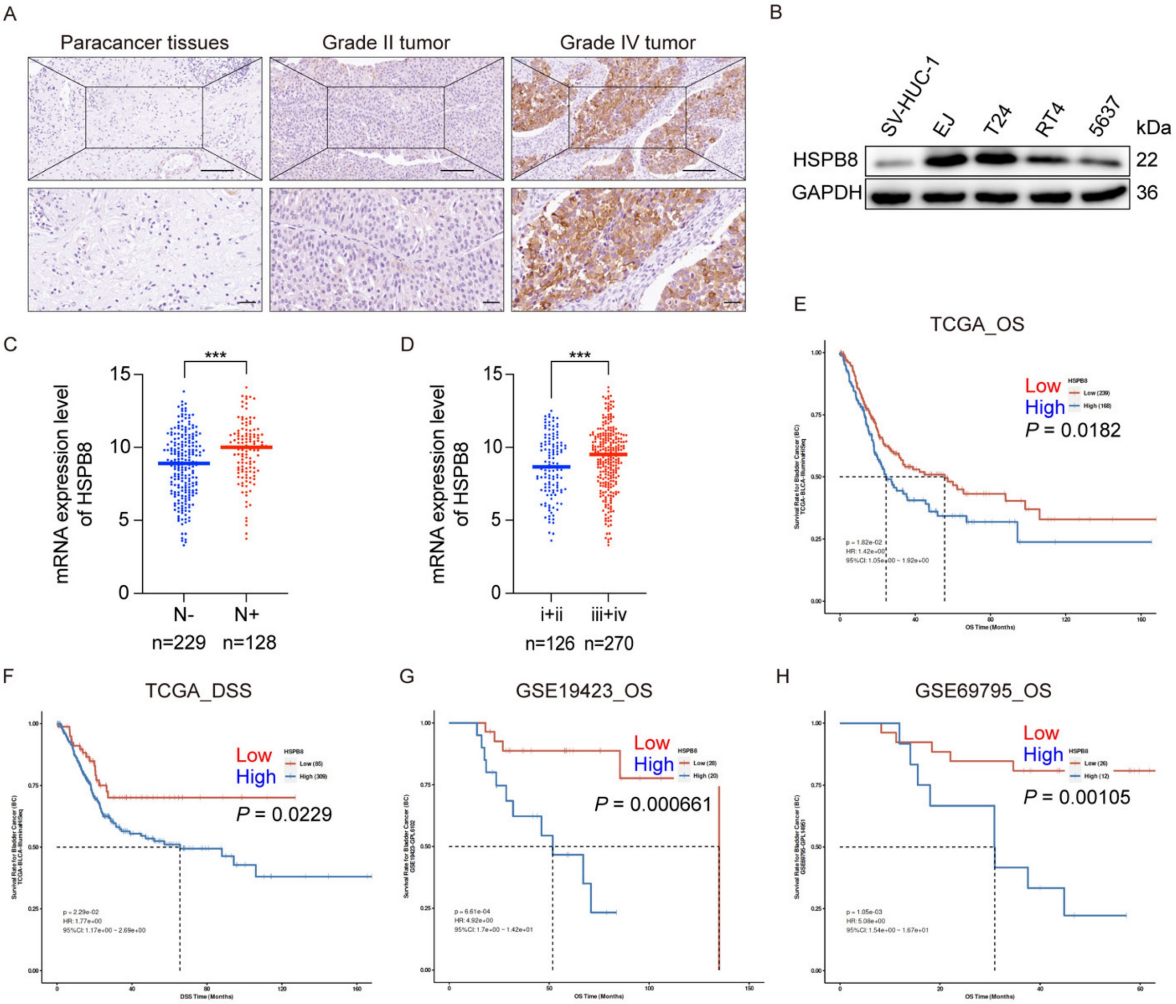
HSPB8 expression was upregulated in BCa cells, and its elevated expression was associated with BCa progression. (A) Immunohistochemical staining was employed to assess the expression level of HSPB8 in BCa tumor tissues compared to adjacent non-tumorous tissues. Notably, HSPB8 exhibited more pronounced expression in high-grade bladder tumors. Scale bar: 200μm. (B) Western blotting revealed enhanced HSPB8 protein expression levels in BCa cell lines, with SV-HUC-1 serving as the control group representing normal bladder epithelium. (C, D) Analysis of HSPB8 normalized expression level in TCGA BCa tissues displayed a significant rise in patients with lymph node metastasis and advanced clinical stages. (E, F) Survival correlation analysis on HSPB8 expression levels in TCGA bladder cancer specimens showed that elevated HSPB8 is linked to poorer prognosis. OS: Overall survival, DSS: Disease Specific Survival. (G, H) Survival analysis from the GEO public database on bladder cancer datasets consistently indicated a significant correlation between high HSPB8 expression and adverse outcomes. * *P* < 0.05, *** *P* < 0.001.

To elucidate the clinical significance of HSPB8 in bladder cancer tissues, we conducted immunohistochemical staining on 68 bladder cancer tissue samples and 40 adjacent normal tissue specimens. Figure 1A displays the results of the IHC assay, which clearly indicated that HSPB8 expression was substantially elevated in bladder cancer (BCa) tissues compared to adjacent normal tissues (*P* < 0.001) (refer to Table 1). Although HSPB8 was expressed at higher levels in high-grade bladder tumors than in low-grade tumors, the difference was not statistically significant. The Western blot findings (Figure 1B) revealed that BCa cell lines expressed HSPB8 at higher levels than nonmalignant urothelial cells. T24 and EJ cell lines, both exhibiting relatively high HSPB8 levels, were selected for further experiments. Based on the Mann-Whitney U analysis, HSPB8 expression showed significant variances across clinical stages (*P* = 0.007). However, other factors maintained consistent baselines (refer to Table 2). Additionally, Spearman rank correlation analysis substantiated a positive correlation between HSPB8 expression and clinical stage (see Table 3). To further validate the conclusions drawn from the IHC assay, we examined the correlation between mRNA expression levels and clinical characteristics in public bladder cancer datasets. According to RNA-seq results from The Cancer Genome Atlas (TCGA), bladder cancer tissues with positive lymph nodes showed significantly elevated HSPB8 expression (Figure 1C). Similarly, higher clinical stages of bladder cancer also displayed augmented HSPB8 expression (Figure 1D). To explore the potential impact of HSPB8 on the prognosis of bladder cancer patients, we utilized the PanCanSurvPlot 9 online tool to conduct survival analyses on tissue samples from both TCGA and GEO databases. The findings revealed that patients with high HSPB8 expression in the TCGA database had a significantly lower overall survival rate (*P* < 0.05) and disease-specific survival (*P* < 0.05). Similarly, various GEO datasets (GSE19423, GSE69795) 10, 11 consistently showed that patients with high HSPB8 expression had a significantly reduced overall survival rate (*P* < 0.01). In conclusion, our findings suggest that HSPB8 plays a potential role in the onset and progression of bladder cancer.

**Figure 2:**
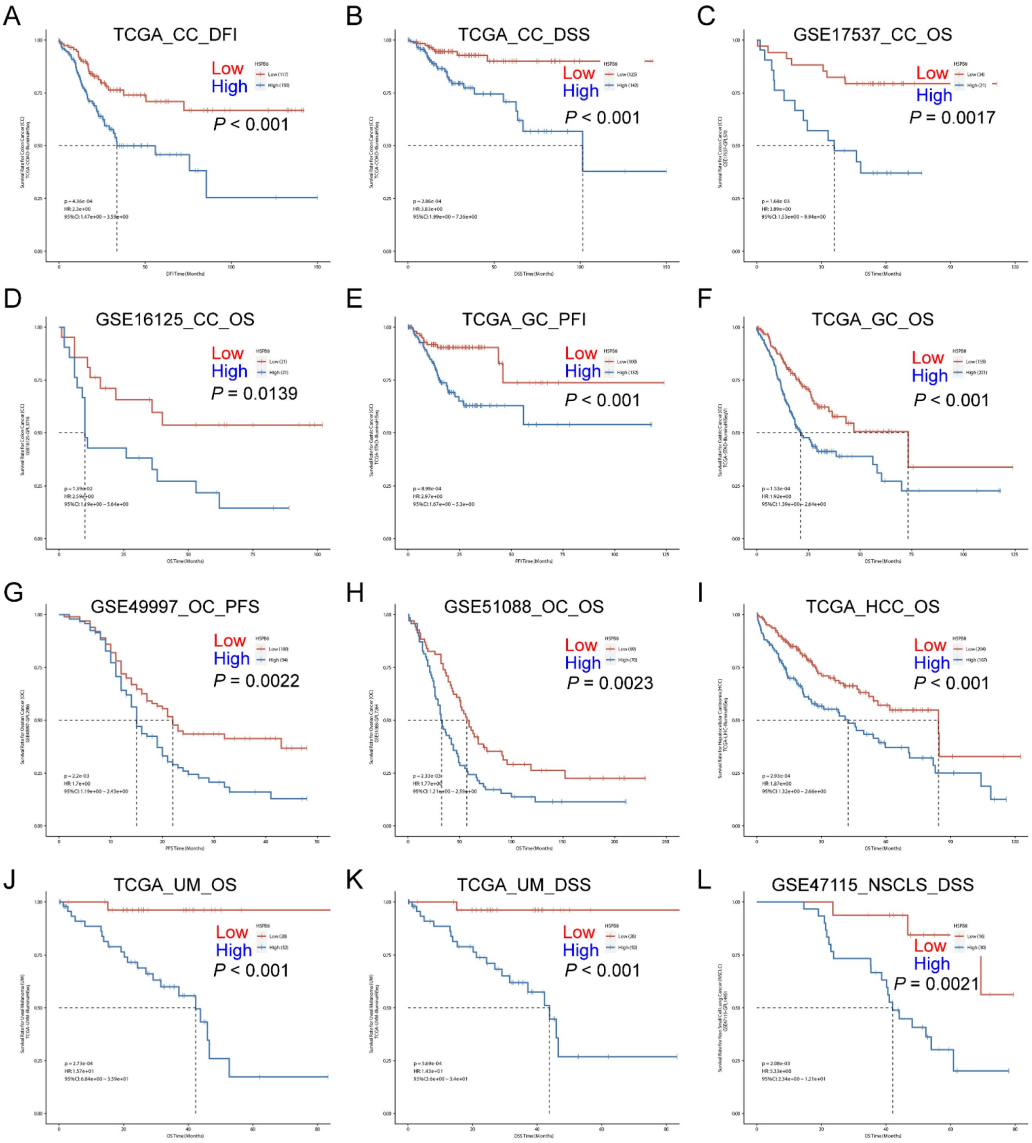
High expression of HSPB8 is associated with poor prognosis in various types of cancer. (A-D) Survival analysis in relation to HSPB8 expression levels in colon cancer public datasets. A and B are derived from the TCGA database, while C and D are from the GEO database. (E, F) Survival analysis based on HSPB8 expression levels for gastric cancer in the TCGA database. (G, H) Survival analysis of HSPB8 in the ovarian cancer dataset from the GEO database. (I) Survival analysis of HSPB8 in the hepatocellular carcinoma dataset in the TCGA database. (J, K) Survival analysis of HSPB8 in the uveal melanoma dataset from the TCGA database. (L) Survival analysis of HSPB8 in the non-small cell lung cancer dataset from the TCGA database. DFI: Disease Free Interval, DFS: Disease Free Survival, OS: Overall Survival, PFI: Progression Free Interval, PFS: Progression Free Survival, DSS: Disease Specific Survival, CC: Colon Cancer, GC: Gastric Cancer, OC: Ovarian Cancer, HCC: Hepatocellular Carcinoma, UM: Uveal Melanoma, NSCLC: Non-Small Cell Lung Cancer.

In addition to the above findings, we also investigated the impact of HSPB8 on patient prognosis in other tumor tissues. We found that high expression of HSPB8 is significantly associated with poorer prognosis in various epithelial tumors (GSE17537, GSE16125, GSE51088, GSE49997, GSE47115) 12-16. This includes colon cancer (Figure 2A-D), gastric cancer (Figure 2E, F), ovarian cancer (Figure 2G, H), hepatocellular carcinoma (Figure 2I), uveal melanoma (Figure 2J, K), and non-small cell lung cancer (Figure 2L).

### Establishment of HSPB8 Knockdown Cell Lines

**Figure 3:**
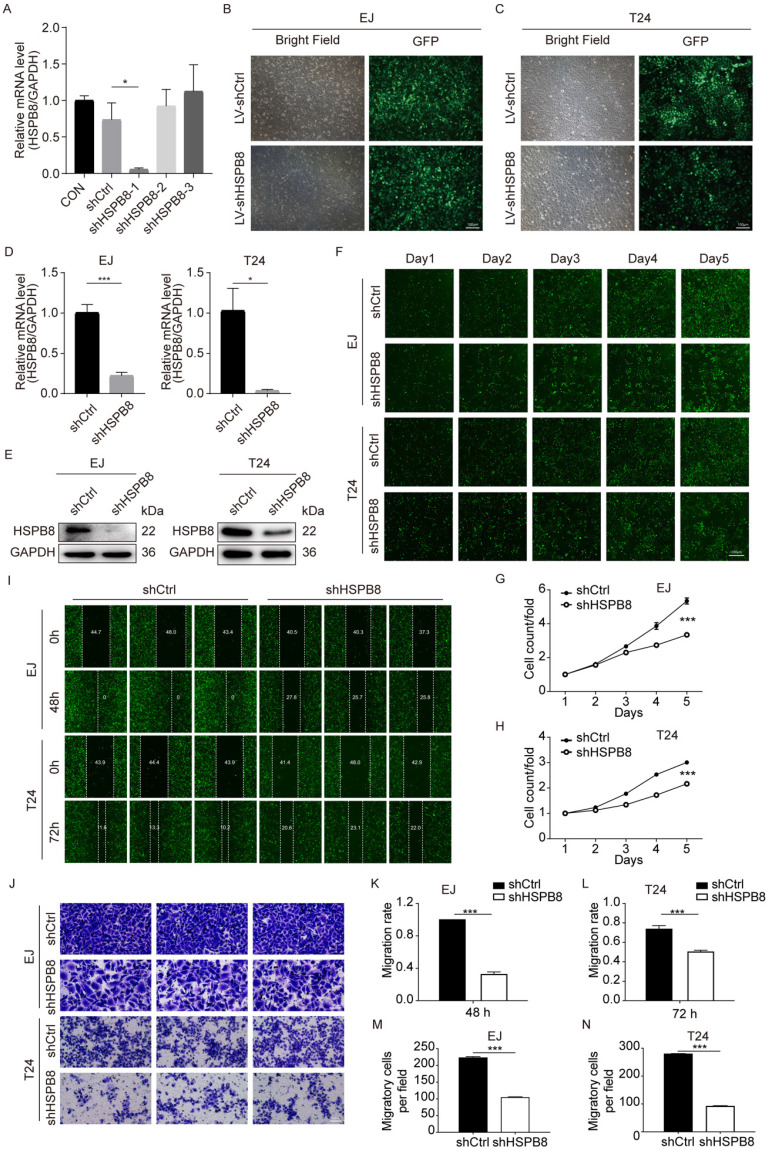
HSPB8 knockdown suppressed cell proliferation and migration in BCa cell lines. (A) The transient knockdown efficiencies of the shHSPB8-1, shHSPB8-2, and shHSPB8-3 groups were confirmed via qRT-PCR, with shHSPB-1 displaying the most effective knockdown. (B-C) To confirm the infection efficiencies of the virus packaged with the shHSPB-1 vector and control vector, the fluorescence of the green fluorescent protein in cells was observed after 72 hours of transfection in EJ and T24 cell lines. Scale bar: 100μm. (D, E) To verify the stable knockdown efficiency of HSPB8, expression levels in lentivirus-infected EJ and T24 cell lines were assessed using qRT-PCR (D) and western blot (E) techniques, indicating satisfactory knockdown results. (F) Cell proliferation in EJ and T24 cells post-infection was determined using the Celigo cell counting assay, with fluorescence microscopy imaging conducted consecutively for five days. Findings suggest that HSPB8 knockdown inhibits bladder cancer cell growth. Scale bar: 200μm. (G, H) Figures G and H represent the statistical graphs of the proliferation efficiency in EJ and T24 cells, respectively, demonstrating that the downregulation of HSPB8 significantly impedes cell growth. (I) Post-infection, the migration rate of EJ and T24 cells was examined via the wound-healing assay. Results reveal a decreased migration rate in the shHSPB8 group compared to the control group. (K, L) K and L are statistical graphs representing the migration rate for EJ and T24 cells from Figure I, respectively, indicating that the downregulation of HSPB8 significantly curtails bladder cancer cell migration. (J) The migration propensity of BCa cells post-infection was further analyzed using a transwell assay. Scale bar: 100μm. In both EJ (M) and T24 (N) cells, a marked reduction in migration rate was observed in chambers upon HSPB8 knockdown. Results are presented as mean ± SD. *P < 0.05, **P < 0.01, and ***P < 0.001.

To elucidate the functional significance of HSPB8 in bladder cancer (BCa), we generated HSPB8 knockdown cell models. For stable and reliable phenotypic alterations, we employed a lentiviral shRNA vector approach. Out of the three shRNA sequences designed, only shHSPB8-1 demonstrated a robust decrease in HSPB8 levels, achieving a knockdown efficiency of 92.3% (*P* < 0.05) (Figure 3A). Consequently, the shHSPB8-1 sequence was selected for all subsequent experiments and is henceforth referred to as shHSPB8. The successful viral infection was visually confirmed by observing the green fluorescent protein (GFP) under a microscope. Notably, the infection efficiency surpassed 80% in both EJ and T24 cell lines (Figure 3B, C). To further validate the knockdown, we assessed HSPB8 expression levels in both the control and shHSPB8 groups using qRT-PCR and western blot assays. The qRT-PCR results demonstrated a significant reduction in HSPB8 mRNA levels by 77.8% (*P* < 0.001) in EJ cells and 96.4% (*P* < 0.05) in T24 cells from the shHSPB8 group (Figure 3D). These findings were in alignment with the results observed in the western blot analysis (Figure 3E). Collectively, our results confirm the successful establishment of the HSPB8 knockdown cell models, making them suitable for subsequent experiments.

### Inhibition of HSPB8 Suppresses Cell Proliferation and Migration In Vitro

Post-lentiviral transduction, we assessed the impact of HSPB8 on bladder cancer cell proliferation utilizing the Celigo cell counting assay, transwell migration assay, and wound-healing assay. The Celigo assay highlighted a marked decrease in cell proliferation in the shHSPB8 group relative to the shCtrl group across both cell lines (Figure 3F-H). Furthermore, the wound-healing assay (Figure 3I) demonstrated a pronounced reduction in migration rates: 67% for EJ cells at 48 hours and 32% for T24 cells at 72 hours (*P* < 0.001 for both, Figures 3K and 3L respectively). In congruence, the chamber transwell assay (Figure 3J) confirmed a diminished migration capability by 53% in EJ cells and 67% in T24 cells upon HSPB8 silencing (*P* < 0.001 for both, Figures 3M and 3N respectively). Collectively, these findings underscore the pivotal role of HSPB8 in regulating the proliferation and migration of bladder cancer cells.

### Silencing of HSPB8 modulates BCa cell cycle dynamics and enhances cellular apoptosis

**Figure 4:**
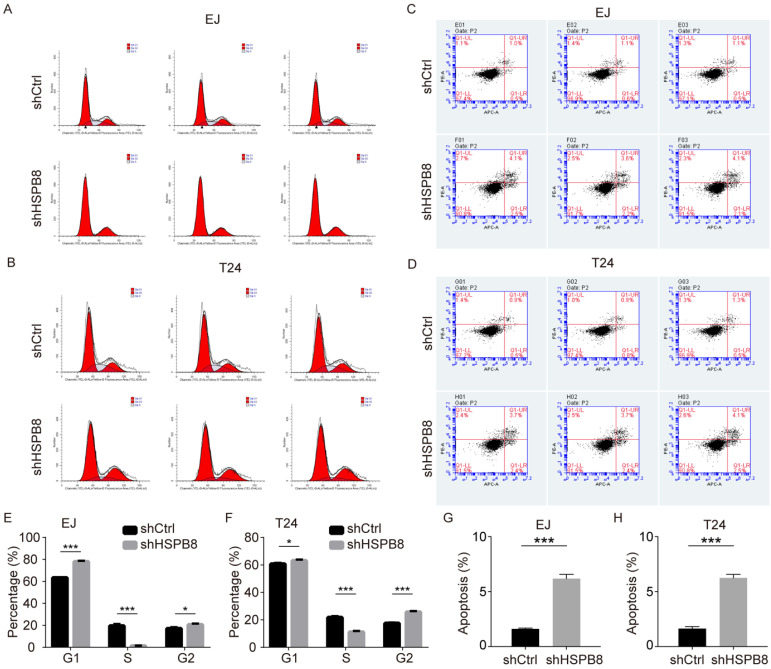
HSPB8 knockdown in bladder cancer cells alters the cell cycle and increases apoptosis. (A, B) Flow cytometry was employed to determine the cell cycle of EJ and T24 cells with or without HSPB8 knockdown. (E, F) Figures E and F are statistical graphs representing the cell cycle of EJ and T24 cells from Figures A and B, respectively. Both indicate a significant reduction in the proportion of cells in the S phase after HSPB8 knockdown. (C, D) The effects of HSPB8 knockdown on apoptosis in EJ and T24 cells were analyzed using flow cytometry. (G, H) Figures G and H are statistical graphs showcasing apoptosis in EJ and T24 cells from Figures C and D, respectively, suggesting a significant increase in apoptosis in bladder cancer cells following HSPB8 knockdown. Results are presented as mean ± SD. *P < 0.05, **P < 0.01, and ***P < 0.001.

Given the observed hindrance in cell growth post-HSPB8 silencing, we postulated this could emanate from disruptions in the cell cycle progression and/or augmented cellular apoptosis. To elucidate this, we undertook flow cytometric evaluations of the cell cycle and apoptosis in BCa cells with HSPB8 knockdown. As delineated in Figure 4A for EJ cells and Figure 4B for T24 cells, a reduced proportion of cells in the S phase was evident with a corresponding pronounced accumulation in the G2 phase (Figure 4E, F). Concurring with our initial hypothesis, apoptosis assays (Figure 4C, D) unveiled a significant escalation in apoptosis rates in the HSPB8-deficient group relative to their control counterparts in both cellular models (*P* < 0.001 for EJ cells, Figure 4G; *P* < 0.001 for T24 cells, Figure 4H).

### Downregulation of HSPB8 Hinders BCa Tumor Expansion In Vivo

**Figure 5:**
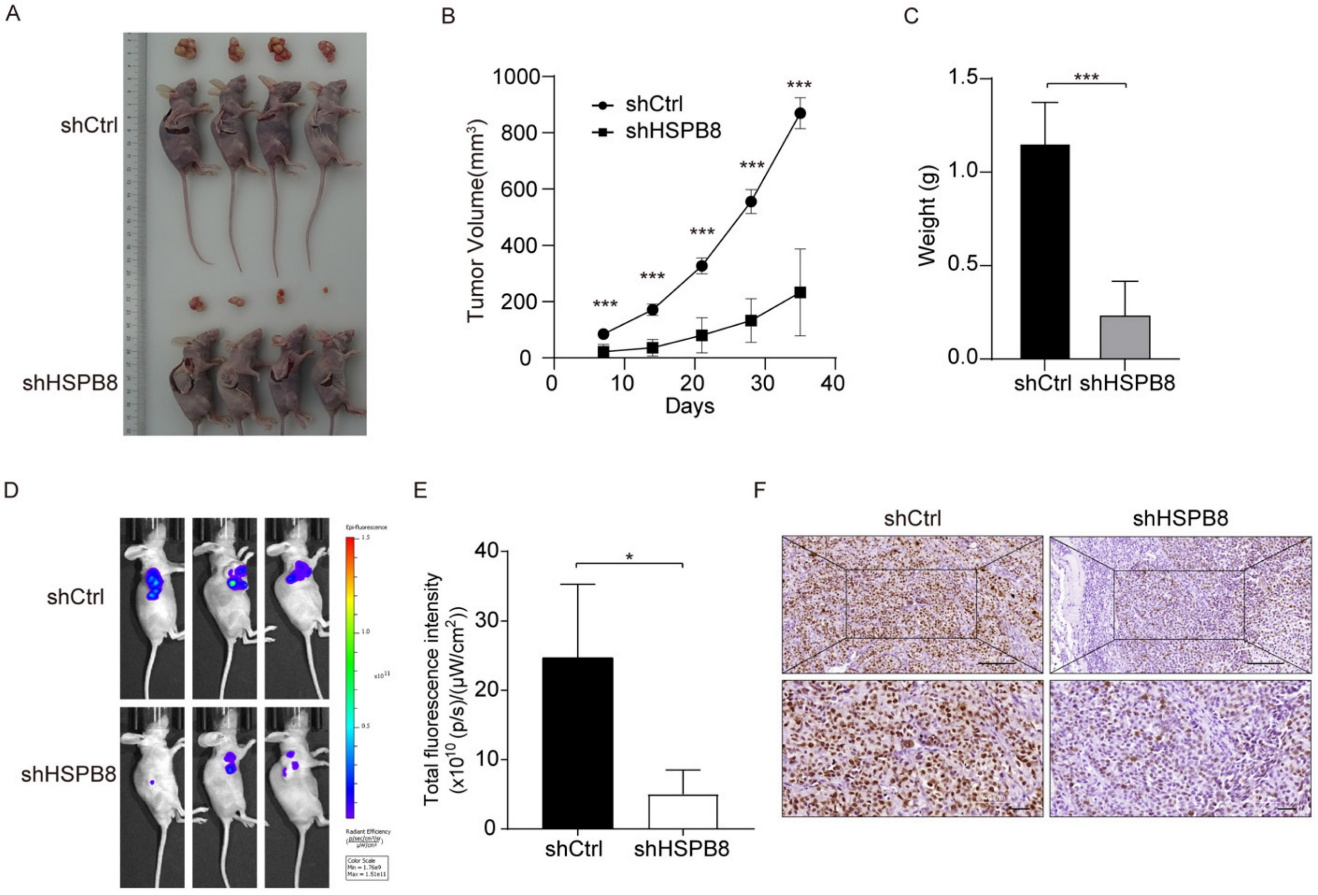
HSPB8 knockdown suppresses BCa tumor growth in vivo. (A) The subcutaneous tumorigenic model in nude mice was employed to assess the impact of HSPB8 on bladder cancer cell growth capability, with the figure illustrating tumor-bearing mice and their corresponding excised tumor tissues. Each group consisted of four mice. (B) Following subcutaneous tumor cell injection, the tumors were measured with calipers every seven days, revealing significant inhibition in the shHSPB8 group. (C) Upon dissection, the subcutaneous tumors were weighed, with tumors from the shHSPB8 group being significantly lighter. (D) Prior to mouse harvesting, in vivo imaging was conducted to assess tumor fluorescence intensity. (E) Figure E represents the statistical analysis of the fluorescence intensity corresponding to Figure D, indicating a significant decrease in the shHSPB8 group. (F) The expression level of Ki-67 was determined via IHC in subcutaneous tumor sections. Scale bar: 200μm. Results are presented as mean ± SD. **P < 0.01 and ***P < 0.001.

To appraise the in vivo ramifications of HSPB8 suppression on BCa growth, we employed xenograft mouse models (Figure 5A). Prior to euthanizing the animals, in vivo fluorescence imaging was utilized to record the tumors' progress (Figure 5D). Post-euthanization, excised tumors were gathered and their weights were ascertained.

Following subcutaneous inoculation of BCa cells, consistent observations were made at various time intervals, showcasing a consistently reduced tumor volume in the shHSPB8 cohort as compared to the shCtrl group (Figure 5B). Quantitative analyses further underscored a significant weight reduction in tumors from the shHSPB8 group (*P* < 0.05, Figure 5C). Diminished fluorescence in the shHSPB8 cohort further accentuated the curtailed tumor expansion, relative to the shCtrl group (Figure 5E, *P* <0.05). Complementing these findings, IHC analyses revealed a subdued Ki67 expression in the shHSPB8 group, further attesting to the attenuated proliferation capabilities of BCa cells upon HSPB8 silencing (Figure 5F). Synthesizing these findings, it becomes evident that HSPB8 depletion exerts a restraining influence on BCa proliferation in a live setting.

### HSPB8 promotes bladder cancer progression via the phosphorylation and activation of HSP27

**Figure 6:**
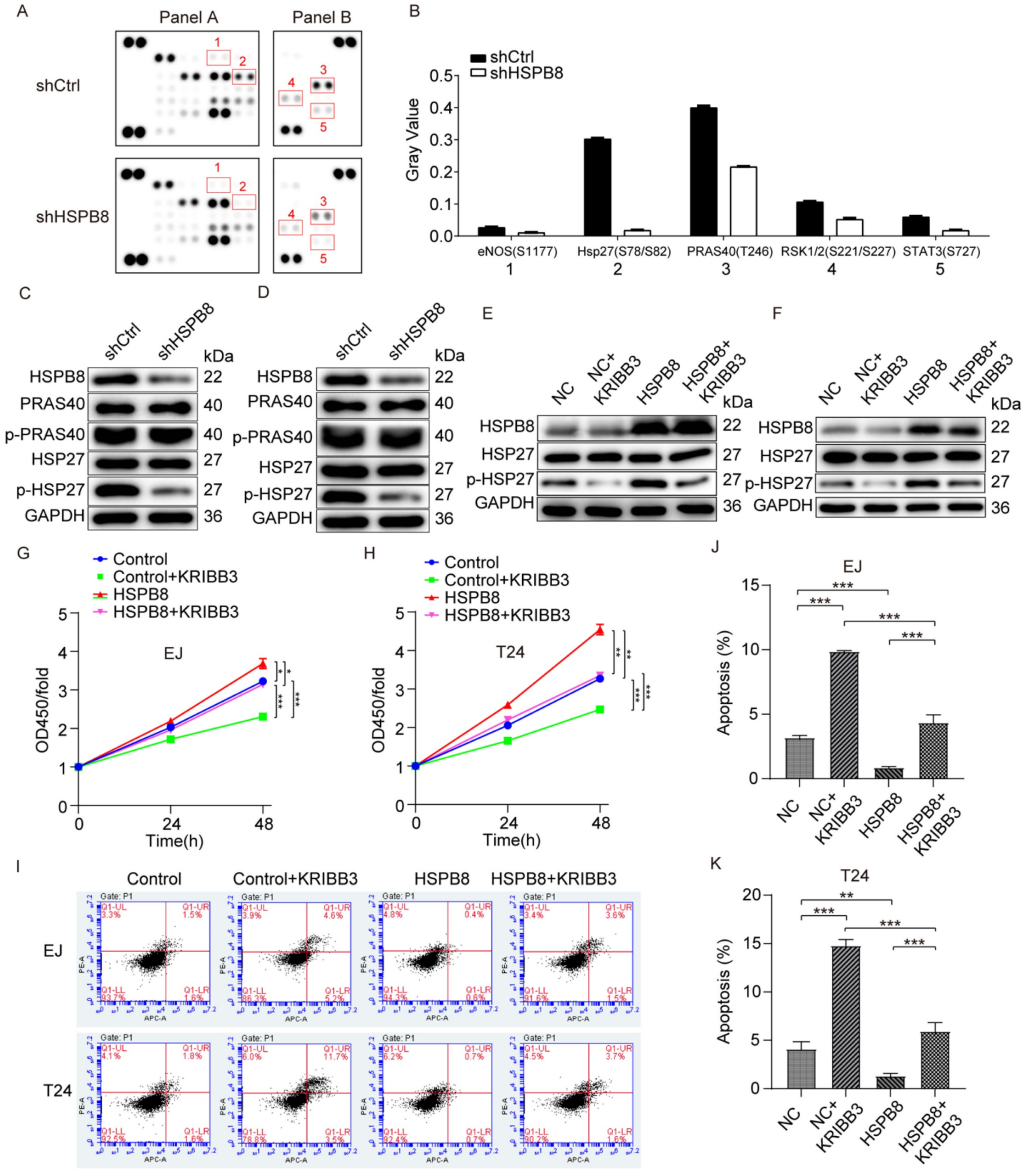
HSPB8 promotes bladder cancer progression via the phosphorylation and activation of HSP27. (A) The expression of phospho-kinases in T24 cells infected with shCtrl and shHSPB8 was determined using the ECL method with Human Phospho-Kinase Array-Membrane. Differentially phosphorylated sites are highlighted with red boxes in the figure. (B) Grayscale quantification of the differential sites observed in (A). Notably, HSP27 (S78/S82) and PRAS40(T246) showed the most pronounced differences between the two groups. (C, D) Western blot analyses were conducted post-HSPB8 knockdown in bladder cancer cells EJ(C) and T24(D) for PRAS40, phosphorylated PRAS40, HSP27, and phosphorylated HSP27 proteins, with the most significant change observed in p-HSP27. (E, F) In bladder cancer cells EJ(E) and T24(F), western blot analyses were performed for the control group, the HSPB8 overexpression group, and the group treated with HSP27 inhibitor KRIBB3 to assess HSP27 and phosphorylated HSP27 proteins. It was confirmed that KRIBB3 can dephosphorylate HSP27. (G, H) CCK8 cell proliferation assays were conducted in EJ(G) and T24(H) cells for the control group, the HSPB8 overexpression group, and the group treated with HSP27 inhibitor. (I-K) Flow cytometry apoptosis assays were performed in EJ and T24 cells for the control group, the HSPB8 overexpression group, and the group treated with the HSP27 inhibitor. Figures J and K represent the corresponding apoptosis statistical analyses for EJ and T24 cells from Figure I. Results are presented as mean ± SD. *P < 0.05, **P < 0.01, and ***P < 0.001.

To initially investigate the mechanism through which HSPB8 modulates BCa cell phenotype, we examined the activity of phospho-kinases due to their pivotal roles in cell growth, apoptosis, and metastasis. The changes in phospho-kinase levels were analyzed using Human Phosphokinase Array-Membranes (Figure 6A). Results from the phospho-protein array suggested that HSPB8 depletion altered the levels of multiple kinases. This included decreased levels of eNOS (S1177), Hsp27 (S78/S82), PRAS40(T246), RSK1/2(S221/S227), and STAT3 (S727), with Hsp27 (S78/S82) and PRAS40(T246) showing the most pronounced reduction (Figure 6B). Subsequent validation in BCa cell lines EJ (Figure 6C) and T24 (Figure 6D) using western blot revealed no significant change in total HSP27 post HSPB8 depletion, though phosphorylated HSP27 levels decreased. Neither total nor phosphorylated PRAS40 protein levels showed notable alterations. To further elucidate the downstream mechanism, we utilized KRIBB3, an inhibitor of HSP27, to validate its inhibitory effect on HSP27 in bladder cancer cells, confirming its suitability for subsequent experiments (Figure 6E, F). To ascertain the impact of HSP27 phosphorylation on HSPB8-mediated regulation of BCa cell functions, we conducted rescue experiments using CCK8 cell proliferation and apoptosis assays. Proliferation assay results revealed a significant enhancement in BCa cell proliferation capability after HSPB8 overexpression. However, upon adding KRIBB3, this enhanced cell proliferation was mitigated (Figure 6G, H). Flow cytometry results for apoptosis indicated a significant suppression in apoptosis rate of BCa cells post HSPB8 upregulation, but the addition of KRIBB3 notably restored the apoptosis rate (Figure 6I-K).

In summary, these results suggest that HSPB8 can activate the HSP27 pathway and regulate the malignant phenotype of BCa cells.

## Discussion

It was estimated that there were 500,000 new cases of BCa and 200,000 deaths worldwide 17. Based on pathological type, this disease can be divided into non-muscle invasive bladder cancer and muscle-invasive bladder cancer. Approximately 30% of BCa is muscle-invasive cancer (MIBC). Despite a variety of treatments being optional, such as surgery, chemotherapy, radiotherapy, and immunotherapy, the outcome for patients with muscle-invasive bladder cancer is far from satisfactory 18. Hence, there is an urgent need to explore the molecular mechanisms underlying BCa development and progression and find more potential therapeutics to improve disease outcomes. Here, we defined for the first time the evidence that HSPB8 was involved in the development and progression of BCa, which might act as a promising therapeutic target for BCa treatment.

Heat shock proteins are a large family of proteins that increase when exposed to environmental stresses. The HSPB family contains HSPB8, which participates in diverse pathological processes, such as neurological diseases 19, 20, myocardial ischemia 21, cancers 22-24, and autoimmune diseases 25. Overexpression of HSPB8 in RBE cells facilitated Vimentin expression and the LC3-II/LC3-I ratio and inhibited E-cadherin and p62 expression, hence promoting intrahepatic cholangiocarcinoma progression by accelerating EMT and autophagy 26. In line with previous findings, the work presented in the paper focused on the potential relationship between HSPB8 and BCa. The results indicated not only that the overexpression of HSPB8 was pronounced in BCa tissues but also that HSPB8 expression was positively correlated with tumor stage. Another study explored HSPB8 expression in bladder cancer and found that its mRNA expression in bladder cancer was lower than in normal epithelial tissues 8, which appears inconsistent with our findings. In our research, we utilized IHC and Western Blot techniques to detect HSPB8 protein expression in bladder tissues from Chinese individuals. The observed inconsistency might be attributed to genetic expression variations among different populations or post-transcriptional modifications 27, 28. Importantly, our study's primary objective was to explore the function exerted by HSPB8 in tumor cells. We found a positive correlation between tumor progression indicators, such as staging, and HSPB8 expression levels, both in our tissue samples and in international public databases. Furthermore, our statistical analysis across multiple public database datasets revealed a significant association between high HSPB8 expression and poorer prognosis in various cancers, including bladder cancer, suggesting HSPB8's potential oncogenic function. Functionally, our findings show that HSPB8 knockdown in BCa cells inhibited cell proliferation and migration, arrested cell cycle progression, and induced cell apoptosis in vitro. Mechanistically, the results of the Human Phospho-Kinase Array-Membrane demonstrated downregulated levels of eNOS (S1177), Hsp27 (S78/S82), PRAS40 (T246), RSK1/2 (S221/S227), and STAT3 (S727) after HSPB8 knockdown. Notably, Hsp27 (S78/S82) and PRAS40 (T246) exhibited the most significant reduction. Western blot results verified that HSPB8 knockdown curbed phospho-HSP27 protein expression. The RSK family is a group of Ser/Thr protein kinases that participate downstream of the MAPK signaling pathway. The expression of the RSK family is usually dysregulated in various types of cancer. It is generally thought that RSK1 and RSK2 promote cancer cell growth 29, survival, and proliferation, while RSK3 may function as a tumor suppressor 30.

KRIBB3 (5-(5-ethyl-2-hydroxy-4-methoxyphenyl)-4-(4-methoxyphenyl)isoxazole) is a chemical compound identified as a potent and specific inhibitor of Heat Shock Protein 27 (HSP27) 31. Interestingly, using an HSP27 inhibitor counteracted the proliferation and apoptosis phenotype induced by elevated HSPB8 expression. Based on the above results, we concluded that HSPB8 knockdown impaired the development and progression of BCa via deactivating HSP27.

Heat Shock Protein 27 (HSP27), encoded by the HSPB1 gene, is a member of the small heat shock protein (sHSP) family. Like its counterparts in the heat shock protein family, HSP27 responds to a variety of stressors, including oxidative stress, anticancer agents, and, notably, elevated temperatures 32. Its phosphorylation at specific serine residues (Ser15, Ser78, and Ser82) is mediated by the action of mitogen-activated protein kinase-activated protein kinase-2 (MAPKAPK-2), an effector of the p38 MAPK pathway. This phosphorylation induces a structural change in HSP27, transforming it from large oligomers into smaller, more functional units 33.

In the context of bladder cancer, early research has found that knocking down HSP27 expression levels in its cell lines can induce apoptosis, augment chemosensitivity, and inhibit tumor growth in mice. This emphasizes the crucial role of HSP27 in bladder cancer dynamics and therapeutic susceptibilities 34. Expanding on this foundation, several studies have underscored HSP27's pivotal role in promoting cancer progression, notably in cancers of the prostate and stomach 35, 36. Furthermore, HSP27 has been discerned to neutralize key apoptotic agents such as cytochrome c and DAXX, thereby thwarting the subsequent activation of caspases 37. HSP27 also engages with reactive oxygen species, mitigating oxidative damage that could otherwise instigate tumor cell death 38.

Structurally, HSP27 features a C-terminal α-crystallin domain and an N-terminal region. Upon phosphorylation, the N-terminus can further mediate the functions of the C-terminal domain, leading to dimer formation 39. Research by Rainer et al., utilizing a yeast two-hybrid approach, identified that the triple aspartate form of HSP27 favors binding within the HSP protein family, whereas the wild-type HSP27 struggles to bind to HSPB8 40. Intriguingly, some reports suggest that HSP27 can amplify breast cancer progression by bolstering the SUMOylation of HSPB8, which in turn enhances its protein stability 41. This suggests a potential mutual regulatory dynamic between HSPB8 and HSP27, emphasizing the need for further comprehensive studies to uncover potential therapeutic avenues in cancer treatment.

## Conclusion

In conclusion, these results indicated that HSPB8 played a remarkable role as a tumor promotor and might be regarded as a promising therapeutic target for more effective treatment of BCa. More in-depth regulatory mechanisms are required to be explored to support the role of HSPB8 in BCa.

## Figures and Tables

**Table 1:** Expression patterns in bladder cancer tissues and para-carcinoma tissues revealed in immunohistochemistry analysis

HSPB8 expression	Tumor tissue		Para-carcinoma tissue		p value
	Cases	Percentage	Cases	Percentage	0.000
Low	28	42.4%	36	100%	
High	38	57.6%	0	-	

**Table 2:** Relationship between HSPB8 expression and tumor characteristics in patients with bladder cancer

	No. of patients	HSPB8 expression		p value
		Low	High	
All patients	66	28	38	
Age (years)				0.176
<71	32	16	16	
≥71	33	11	22	
Gender				0.226
Male	56	22	34	
Female	10	6	4	
Tumor size				0.754
≤4cm	34	15	19	
>4cm	25	10	15	
Grade				0.595
I	2	1	1	
II	4	2	2	
III	6	2	4	
Stage				0.007
1	6	3	3	
2	13	11	2	
3	21	7	14	
4	13	3	10	
T Infiltrate				0.096
T1	11	4	7	
T2	16	13	3	
T3	25	6	19	
T4	5	2	3	
Lymphatic metastasis (N)				0.335
0	47	22	25	
1	10	3	7	
Positive lymph node number				0.313
=0	44	21	23	
>0	10	3	7	

**Table 3:** Relationship between HSPB8 expression and tumor characteristics in patients with bladder cancer

		HSPB8
Stage	Spearman correlation	0.373
	Signification (double-tailed)	0.006
	N	53
